# Survival of salivary gland cancer stem cells requires mTOR signaling

**DOI:** 10.1038/s41419-021-03391-7

**Published:** 2021-01-21

**Authors:** Nathalia P. Andrade, Kristy A. Warner, Zhaocheng Zhang, Alexander T. Pearson, Andrea Mantesso, Douglas M. Guimaraēs, Albina Altemani, Fernanda V. Mariano, Fabio D. Nunes, Jacques E. Nör

**Affiliations:** 1 Department of Periodontics and Oral Medicine, University of Michigan, Ann Arbor, MI USA; 2 Department of Oral Pathology, University of São Paulo, São Paulo, SP Brazil; 3 Department of Cariology, Restorative Sciences, Endodontics, University of Michigan, Ann Arbor, MI USA; 4 Department of Pathology, UNICAMP, Piracicaba, SP Brazil; 5 Department of Otolaryngology, University of Michigan, Ann Arbor, MI USA; 6 University of Michigan Rogel Comprehensive Cancer Center, Ann Arbor, MI USA

**Keywords:** Cancer stem cells, Cancer stem cells, Head and neck cancer, Oral cancer

## Abstract

Advanced salivary gland mucoepidermoid carcinoma (MEC) is a relentless cancer that exhibits resistance to conventional chemotherapy. As such, treatment for patients with advanced MEC is tipically radical surgery and radiotherapy. Facial disfigurement and poor quality of life are frequent treatment challenges, and many patients succumb to loco-regional recurrence and/or metastasis. We know that cancer stem-like cells (CSC) drive MEC tumorigenesis. The current study tests the hypothesis that MEC CSC are sensitive to therapeutic inhibition of mTOR. Here, we report a correlation between the long-term clinical outcomes of 17 MEC patients and the intratumoral expression of p-mTOR (*p* = 0.00294) and p-S6K1 (*p* = 0.00357). In vitro, we observed that MEC CSC exhibit constitutive activation of the mTOR signaling pathway (i.e., mTOR, AKT, and S6K1), unveiling a potential strategy for targeted ablation of these cells. Using a panel of inhibitors of the mTOR pathway, i.e., rapamycin and temsirolimus (mTOR inhibitors), buparlisib and LY294002 (AKT inhibitors), and PF4708671 (S6K1 inhibitor), we observed consistently dose-dependent decrease in the fraction of CSC, as well as inhibition of secondary sphere formation and self-renewal in three human MEC cell lines (UM-HMC-1,-3A,-3B). Notably, therapeutic inhibition of mTOR with rapamycin or temsirolimus induced preferential apoptosis of CSC, when compared to bulk tumor cells. In contrast, conventional chemotherapeutic drugs (cisplatin, paclitaxel) induced preferential apoptosis of bulk tumor cells and accumulation of CSC. In vivo, therapeutic inhibition of mTOR with temsirolimus caused ablation of CSC and downregulation of Bmi-1 expression (major inducer of stem cell self-renewal) in MEC xenografts. Transplantation of MEC cells genetically silenced for mTOR into immunodeficient mice corroborated the results obtained with temsirolimus. Collectively, these data demonstrated that mTOR signaling is required for CSC survival, and unveiled the therapeutic potential of targeting the mTOR pathway for elimination of highly tumorigenic cancer stem-like cells in salivary gland mucoepidermoid carcinoma.

## Introduction

Mucoepidermoid carcinoma (MEC) is considered the most common malignant tumor arising from salivary glands, accounting for approximately one third of all salivary gland malignancies^1^. MEC is known as a malignancy that exhibits diverse biological and clinical behaviors ranging from highly aggressive tumors, with great potential for recurrence and metastasis, to tumors that demonstrate a more benign nature^2^. Surgical resection is the main therapeutic method for MEC treatment, leading to esthetic complications and facial disfigurement in many patients^3^. In advanced and highly aggressive cases, treatment includes radiotherapy, which is not very effective at preventing loco-regional recurrence and distant metastasis^4^. Platinum-based drugs typically have modest benefits, as this cancer is largely resistant to chemotherapy^3^. Improving the understanding of the pathobiology of MEC is essential for the identification of novel targets therapies to overcome drug resistance and to improve patient outcome.

Recent studies have focused on the development of better systemic therapies for MEC^5^. However, most of these studies do not take into account the function of cancer stem-like cells (CSC). These cells constitute a rare, self-renewing population of highly tumorigenic cells comprising less than 10% of the total cells in the tumor^6^. It has been shown that CSC drive metastasis and recurrence in several cancer types^7,8^. Considering that CSC are typically resistant to chemotherapy and radiotherapy, these cells may be involved in the resistance of MEC to cytotoxic and radiotherapy^9,10^. Our laboratory identified the presence and function of CSC in MEC using high ALDH activity and CD44 expression as markers for this population^11^. It has been shown in some solid tumors that inhibition of the PI3K-mTOR signaling pathway may induce tumor cell differentiation^12,13^, but the impact of therapeutic blockade on MEC CSCs is unknown.

The PI3K/AKT signaling cascade is frequently upregulated in human cancers, causing hyperactivation of the Mammalian target of rapamycin (mTOR) pathway^14^. Indeed, aberrant activation of mTOR has been observed in salivary gland tumors^15–17^. mTOR is a central regulator of multiple cellular processes promoting cancer cell growth, survival, and metastasis^18^. Two distinct complexes are formed by the interaction of mTOR with other proteins, the mTOR complex 1 (mTORC1) and mTOR complex 2 (mTORC2)^19^. These complexes have distinct functions, substrates, sensitivity to rapamycin (mTOR inhibitor) and form networks of interactions and feedback loops with other signaling molecules within the PI3K/AKT pathway^20^. mTORC1 mediates protein translation and cell growth through phosphorylation of its downstream targets, i.e., ribosomal S6 protein kinase 1 (S6K1) and eIF4E-binding protein 1 (4E-BP1)^21^. In contrast, mTORC2 plays an important role in cell survival, metabolism, proliferation, and cytoskeleton organization^22,23^.

As mTOR plays a major role in carcinogenesis, there is much interest in mTOR inhibitors as potential anticancer agents. It is possible to interfere in mTOR activity through the use of specific inhibitors, such as rapamycin (sirolimus) or rapamycin analogs (rapalogs)^24^. Rapalogs (e.g., temsirolimus, everolimus) inhibit mTOR through the same mechanism as rapamycin, however they exhibit better pharmacokinetic properties^25^. Temsirolimus and everolimus are FDA-approved for the treatment of renal cell carcinoma. Likewise, everolimus has been FDA-approved for neuroendocrine tumors and metastatic breast cancer treatments^26,27^. Indeed, several ongoing clinical trials are testing the safety and efficacy of rapalogs as therapy, alone or in combination with conventional chemotherapy agents, for treatment of other cancer types^28^. However, the effect of therapeutic inhibition of mTOR on CSCs in salivary gland MEC remains unclear.

Considering the involvement of mTOR signaling in carcinogenesis and the observation that the mTOR pathway is constitutively active in MEC CSC, we hypothesized that therapeutic inhibition of mTOR may constitute a viable approach to eliminate CSC. Here, we observed that therapeutic inhibition of mTOR reduces the CSC fraction in MEC by blocking their self-renewal and inducing apoptosis. We also observed that inhibition of mTOR caused tumor regression and reduced tumor vascular density in short-term experiments performed in xenograft models of MEC.

## Results

### mTOR pathway is constitutively active in MEC cancer stem-like cells

**Fig. 1:**
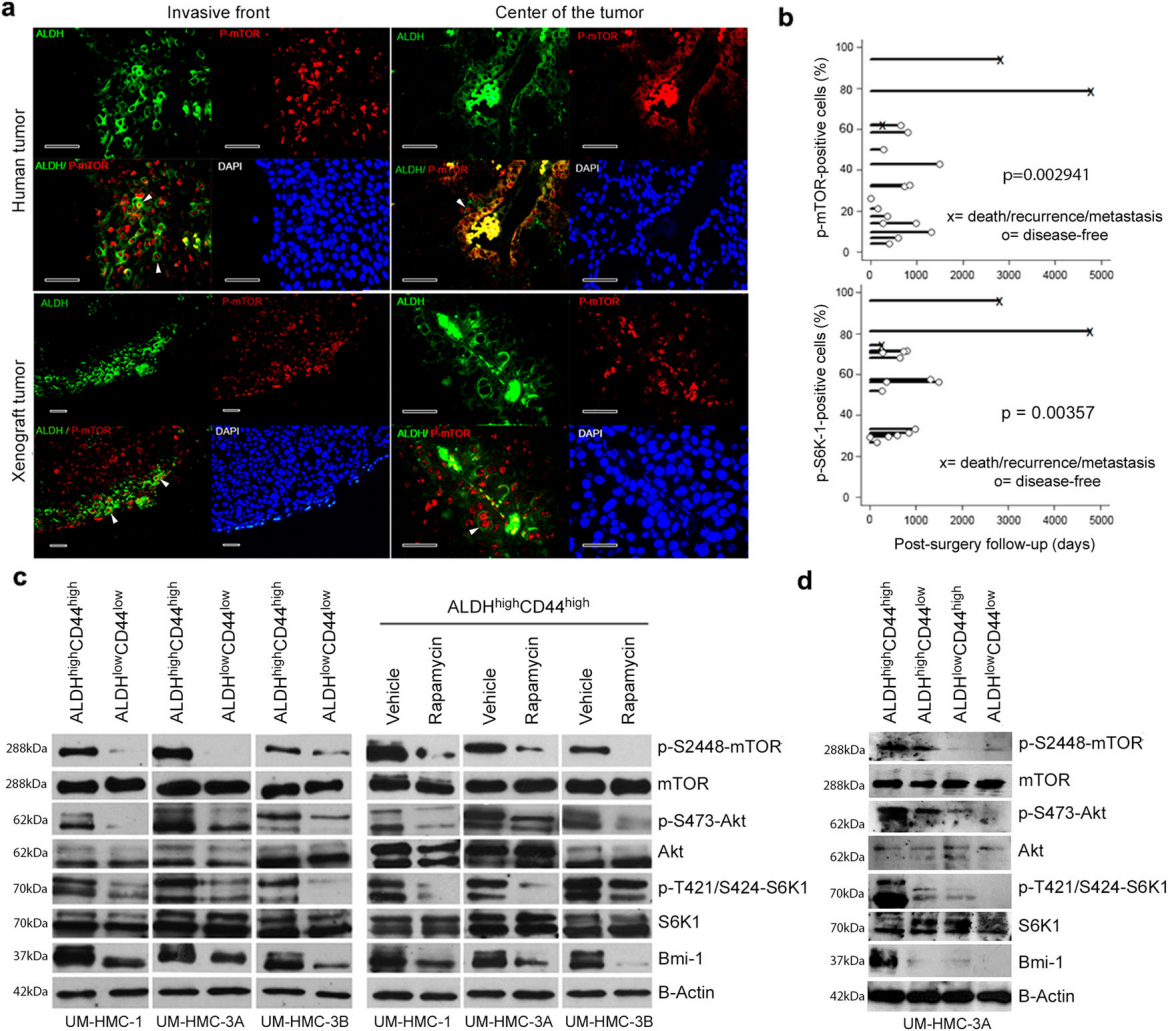
mTOR pathway is constitutively active in cancer stem-like cells in human mucoepidermoid carcinomas. **a** Immunofluorescence staining of primary human salivary gland mucoepidermoid carcinoma or xenograft tumors generated from UM-HMC-3A cells. ALDH1 is stained in green, p-mTOR is stained in red and DAPI is stained in blue. Scale bars represent 25 µm. **b** Graphs depicting the correlation of survival of MEC patients after surgery and expression of pS6K1 and p-mTOR (*n* = 17; five fields per slide). **c** Western blots for AKT, p-S473-AKT, mTOR, p-S2448-mTOR, S6K1, p-T421/S424-S6K1, and Bmi-1 in ALDH^high^CD44^high^ or ALDH^low^CD44^low^ cells sorted from UM-HMC-1, UM-HMC-3A or UM-HMC-3B cells. Alternatively, ALDH^high^CD44^high^ cells (i.e., cancer stem-like cells) were exposed to 2 ng/ml rapamycin or vehicle control for 24 h. **d** Western blots for AKT, p-S473-AKT, mTOR, p-S2448-mTOR, S6K1, p-T421/S424-S6K1, Bmi-1 in ALDH^high^CD44^high^, ALDH^high^CD44^low^, ALDH^low^CD44^high^, or ALDH^low^CD44^low^ cells sorted from UM-HMC-3A cells. Experiments were performed three independent times to verify reproducibility of the data.

To evaluate the patterns of expression of ALDH1 (CSC marker) and p-mTOR in human MEC, we performed immunofluorescence studies in tumor specimens retrieved from patients or in xenograft tumors generated in mice (Fig. 1a). We observed that ALDH1-positive cells were found primarily in the invasive fronts or in close proximity to blood vessels, reproducing what we observed previously in head and neck squamous cell carcinomas (HNSCC)^29^. While ALDH1 was expressed primarily in the cytoplasm, p-mTOR was found in both, nucleus and cytoplasm (Fig. 1a). Immunofluorescence performed in xenograft tumors generated by UM-HMC-3A cells corroborated the primary human tumor data, and indicated that most ALDH1-positive cells also express p-mTOR. To correlate the expression of p-mTOR and p-S6K1 with the outcomes of patients with MEC, we performed immunohistochemical analyses of tissues collected from 17 patients followed-up for up to 13 years (Fig. 1b and Supplementary Fig. 1). Despite the fact that we had a relatively small number of patients with sufficient follow-up (rare disease), we observed a correlation between number of p-mTOR-positive cells and incidence/timing of metastasis, recurrence, or death (*p* = 0.002941). Data from the p-S6K1 analysis corroborated these results, showing a positive correlation between patient outcomes and p-S6K1 expression (*p* = 0.00357) (Fig. 1b).

Then, we performed flow cytometry-based cell sorting in three human MEC cell lines (UM-HMC-1, UM-HMC-3A, and UM-HMC-3B) to identify the CSC subpopulation (i.e., ALDH^high^C44^high^ cells), and correlate it with activity of the mTOR pathway (Fig. 1c). We observed that ALDH^high^CD44^high^ cells exhibit constitutive phosphorylation of mTOR, AKT, S6K1, and expression of Bmi-1 (marker of self-renewal), when compared with control ALDH^low^CD44^low^ cells (Fig. 1c). Interestingly, ALDH^high^CD44^low^ cells presented lower activity of the mTOR pathway and expression of Bmi-1 (when compared to ALDH^high^CD44^high^ cells), and ALDH^low^CD44^high^ showed minimal (or no) phosphorylation events and expression of Bmi-1 (levels comparable to ALDH^low^CD44^low^ cells), indicating a correlation between ALDH activity (and to a lesser extend CD44 expression) and activation of the mTOR pathway (Fig. 1d). To evaluate the impact of mTOR inhibitors in MEC CSC, we exposed ALDH^high^CD44^high^ cells to rapamycin for 24 h. Rapamycin effectively inhibited AKT, mTOR, and S6K1 phosphorylation, as well as Bmi-1 expression in ALDH^high^CD44^high^ cells (Fig. 1c). Collectively, these results provided the scientific rationale for a more in-depth pursue of the impact of mTOR inhibition on MEC stemness.

### Inhibition of mTOR signaling reduces the fraction and self-renewal of CSC

To evaluate the therapeutic potential of inhibitors of the mTOR signaling pathway in MEC, we performed time-dependence and dose-dependence assays for buparlisib (AKT inhibitor), rapamycin, and temsirolimus (mTOR inhibitors), and PF4708671 (S6K1 inhibitor) using the SRB assay (Supplementary Fig. 2a). We observed a dose-dependent and time-dependent cytotoxic response in all MEC cells lines evaluated. The IC_50_ values for each one of these agents were achieved in low concentrations of the inhibitors, evidencing the importance of mTOR signaling pathway in the survival of MEC cells.

**Fig. 2:**
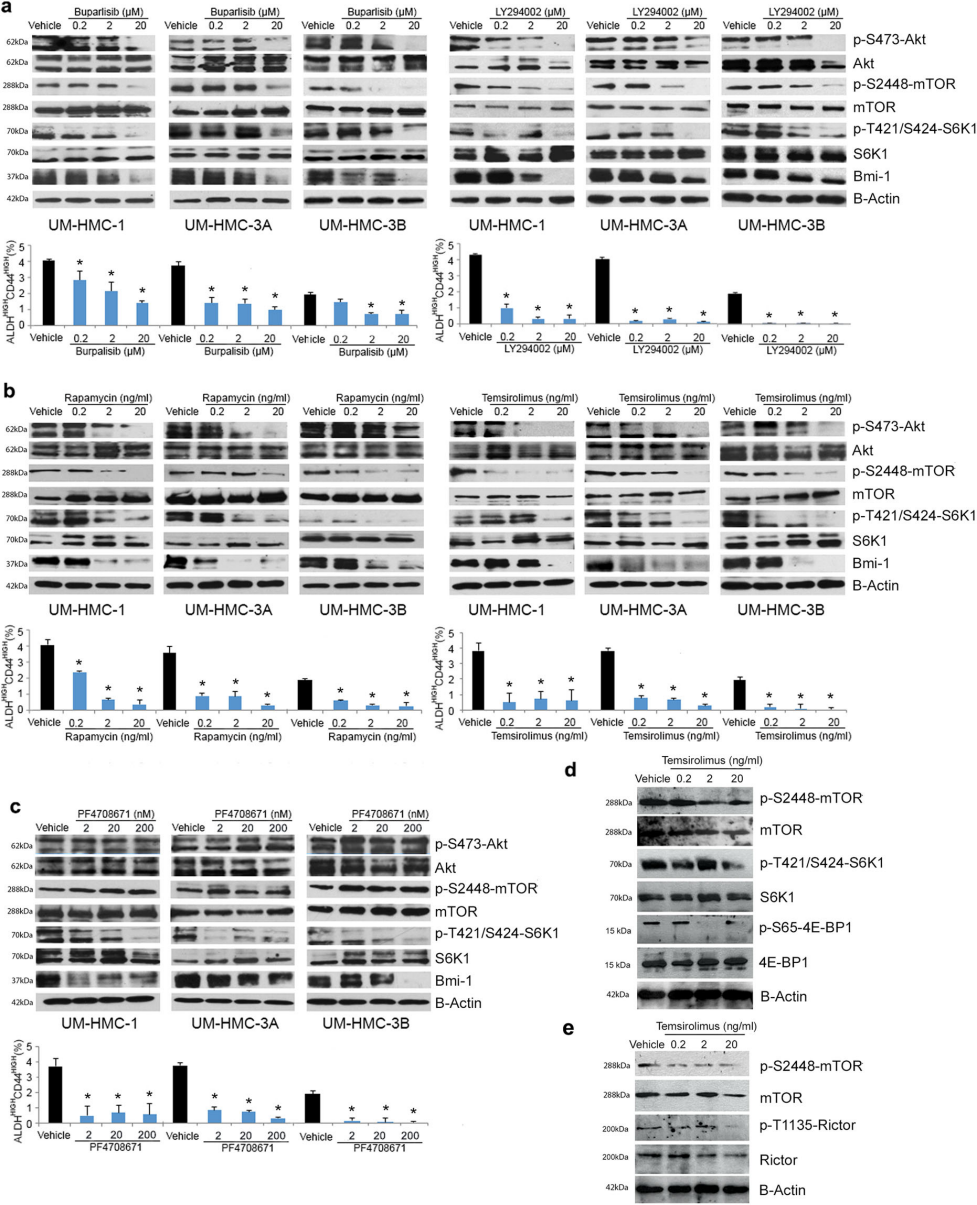
Inhibition of mTOR signaling reduces the fraction of cancer stem-like cells in human mucoepidermoid carcinoma cells. **a** Western blots for AKT, p-S473-AKT, mTOR, p-S2448-mTOR, S6K1, p-T421/S424-S6K1, Bmi-1, and B-actin after exposure of bulk MEC tumor cells (UM-HMC-1, UM-HMC-3A, and UM-HMC-3B) to increasing concentrations of AKT inhibitors (0.2–20 μM buparlisib or LY2940002) or vehicle control. Graphs depict the fraction of ALDH^high^CD44^high^ cells identified by flow cytometry in MEC tumor cells (UM-HMC-1, UM-HMC-3A, and UM-HMC-3B) after exposure to increasing concentrations of buparlisib, LY2940002, or vehicle control. **b** Western blots for AKT, p-S473-AKT, mTOR, p-S2448-mTOR, S6K1, p-T421/S424-S6K1, and Bmi-1 after exposure of bulk MEC tumor cells to increasing concentrations of mTOR inhibitors (0.2–20 ng/ml rapamycin or temsirolimus) or vehicle control. Graphs depict the fraction of ALDH^high^CD44^high^ cells identified by flow cytometry in MEC tumor cells after exposure to increasing concentrations of rapamycin or temsirolimus. **c** Western blots for AKT, p-S473-AKT, mTOR, p-S2448-mTOR, S6K1, p-T421/S424-S6K1, and Bmi-1 after exposure of bulk MEC tumor cells to increasing concentrations of S6K1 inhibitor (2–200 nM PF4708671) or vehicle control. Graphs depict the fraction of ALDH^high^CD44^high^ cells identified by flow cytometry in UM-HMC-1, UM-HMC-3A, and UM-HMC-3B cell lines after exposure to increasing concentrations of the S6K1 inhibitor. **d** Western blots for mTOR, p-S2448-mTOR, S6K1, p-T421/S424-S6K1, 4E-BP1, and p-S65-4E-BP1 upon exposure of UM-HMC-3A cells to increasing concentrations of temsirolimus for 24 h. **e** Western blots for mTOR, p-S2448-mTOR, Rictor, and p-T1135-Rictor upon exposure of UM-HMC-3A cells to increasing concentrations of temsirolimus for 24 h. Asterisk depicts *p* < 0.05, as determined by one-way ANOVA followed by post-hoc tests for multiple comparisons between vehicle-treated and cells that were exposed to experimental compounds. Error bars indicate standard deviation (SD). Graphs for impacts of treatment on the fraction of ALDH^high^CD44^high^ cells depict data from four wells per experimental condition. Experiments were performed three independent times to verify reproducibility of the data.

To explore the role of the mTOR pathway in MEC CSC, we performed Western blots from bulk MEC tumor cells treated for 24 h with increasing concentrations of several inhibitors of the mTOR pathway. We observed that both buparlisib and LY2940002 inhibited phosphorylation of AKT, mTOR, and S6K1 (Fig. 2a). Rapamycin and temsirolimus caused a dose-dependent inhibition of the mTOR signaling pathway, including AKT phosphorylation (Fig. 2b). This effect presumably occurred by blocking the mTORC2 feedback loop, as Rictor phosphorylation was also inhibited in MEC cells (Fig. 2e), reproducing results observed in other cell types^30^. PF4708671 inhibited specifically phosphorylation of S6K1, without effects on upstream components of this signaling pathway (AKT and mTOR) (Fig. 2c). Notably, inhibition of the mTOR signaling pathway at every level studied here (i.e., AKT, mTOR, or S6K1) resulted in inhibition of Bmi-1 expression (Fig. 2a–c).

To determine the impact of inhibition of mTOR signaling on the CSC fraction, we analyzed the fraction of ALDH^high^CD44^high^ cells by flow cytometry. We observed a significant reduction in fraction of ALDH^high^CD44^high^ cells after treatment with all inhibitors evaluated here (Fig. 2a–c and Supplementary Figs. 3–5). Interestingly, the S6K1 inhibitor (PF4708671) showed a 96-hour IC_50_ in the low µM range (1.43–1.86 μM), while causing a significant decrease in the fraction of ALDH^high^CD44^high^ cells at much lower concentrations (2–200 nM) after 24 h (Fig. 2c and Supplementary Figs. 2, 5). These results suggest that S6K1 phosphorylation is a critical step in the regulation of Bmi-1 and CSC fraction, as the upstream components of the mTOR pathway are not affected by PF4708671 (Fig. 2a–c). To verify the effect of mTOR inhibition on MEC stemness and self-renewal, we performed sphere assays (i.e., salispheres) using a concentration of each inhibitor that is sufficient to cause a decrease in Bmi-1 expression (Fig. 2a–c). We observed that treatment with each one of inhibitors resulted in a significant reduction in number of primary salispheres (Supplementary Fig. 6a, b). To evaluate the impact of mTOR inhibition on CSC self-renewal, we dissociated and passaged the primary spheres into new low attachment plates to generate secondary spheres. As expected, the number of secondary salispheres derived from treated cells was lower than vehicle controls (Supplementary Fig. 6a, b).

To understand possible mechanisms involved in the inhibition of AKT phosphorylation observed here, we exposed UM-HMC-3A cells to increasing concentrations of temsirolimus. We observed a dose-dependent inhibition of 4E-BP1 (major component of mTORC1 complex) and Rictor (major component of the mTORC2 complex) with increasing concentrations of Temsirolimus (Fig. 2d, e). These data suggested that MEC cell sensitivity to mTOR inhibition is probably due to inhibitory effects in mTORC1 and mTORC2, and absence of a feedback loop that maintains AKT phosphorylation.

### mTOR inhibitors induce preferential apoptosis of cancer stem-like cells

**Fig. 3:**
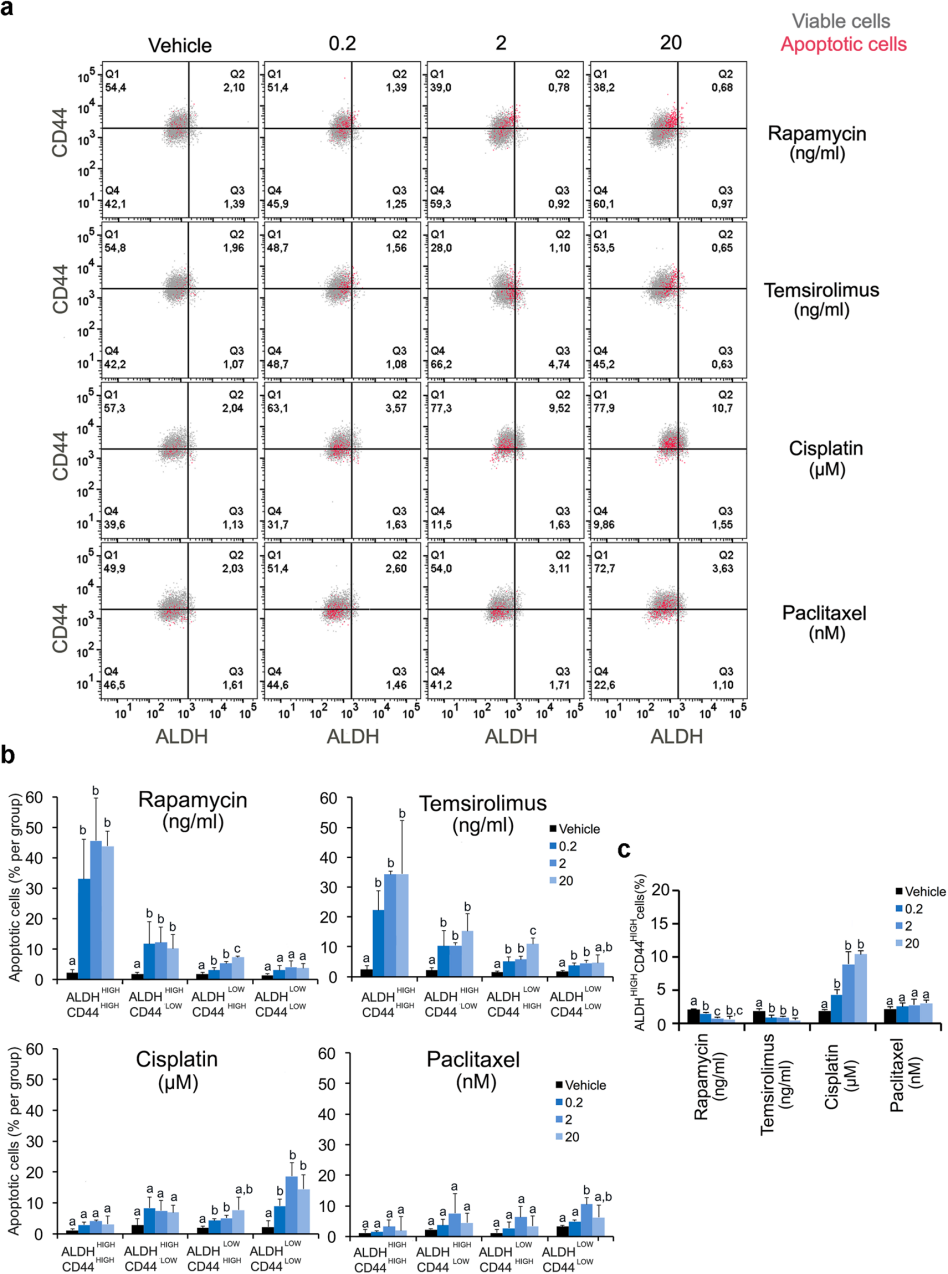
mTOR inhibitors are more effective at inducing apoptosis of cancer stem-like cells than bulk tumor cells. **a** Flow plots for ALDH activity, CD44 expression and Annexin V (early marker of apoptosis) in UM-HMC-3B cells after 24 h of treatment with increasing concentrations of rapamycin, temsirolimus, cisplatin or paclitaxel analyzed by flow cytometry. Viable cells are represented in gray while apoptotic cells (i.e., high expression of Annexin V) are shown in red. **b** Graphs depicting the percentage of apoptotic cells (Annexin V-positive) according to ALDH activity and CD44 expression, i.e., ALDH^high^CD44^high^, ALDH^high^CD44^low^, ALDH^low^CD44^high^, and ALDH^low^CD44^low^ after treatment with Rapamycin, temsirolimus, cisplatin, or paclitaxel. **c** Graph depicting the percentage of ALDH^high^CD44^high^ cells identified by flow cytometry in tumor cells (UM-HMC-3B) treated with increasing concentrations of rapamycin, temsirolimus, cisplatin, or paclitaxel. One-way ANOVA and post-hoc Tukey tests were used all statistical analyses included here. Different low-case letters indicate significant differences among groups (*P* < 0.05), as determined by one-way ANOVA followed by post-hoc Tukey tests for multiple comparisons. Error bars indicate standard deviation (SD). Graphs for impacts of treatment on the fraction of ALDH^high^CD44^high^ or apoptotic cells depict data from four wells per experimental condition. Experiments were performed three independent times to verify reproducibility of the data.

To understand the mechanism that leads to reduction of the fraction of CSCs, we analyzed the expression of Annexin V (early marker of apoptosis), as well as ALDH activity and CD44 expression by flow cytometry in MEC cells treated with mTOR inhibitors (Fig. 3 and Supplementary Figs. 7–9). We also compared the effect of mTOR inhibitors with the effect of chemotherapeutic agents (cisplatin and paclitaxel) on the CSC fraction. mTOR inhibitors (rapamycin and temsirolimus) were highly effective at inducing apoptosis of ALDH^high^CD44^high^ cells (Fig. 3a, b and Supplementary Figs. 8, 9). In contrast, minimal apoptosis was observed in ALDH^low^CD44^low^ exposed to these inhibitors. Further, platinum-based chemotherapeutic agents (i.e., cisplatin and paclitaxel) did not induce apoptosis of ALDH^high^CD44^high^ cells. These drugs were more effective at inducing apoptosis in non-CSC cells, particularly those with low ALDH activity (ALDH^low^CD44^high^ and ALDH^low^CD44^low^ cells). As a consequence of the differential effect of mTOR inhibitors in CSC vs. non-CSC, the overall fraction of CSC decreased upon treatment (Fig. 3c and Supplementary Figs. 8c and 9c). Interestingly, exposure to cisplatin increased the CSC fraction, while exposure to paclitaxel had a more modest effect on these cells (Fig. 3c and Supplementary Figs. 8c and 9c). Together, these results demonstrate that induction of preferential apoptosis of ALDH^high^CD44^high^ cells is a potential mechanism to explain the reduction of CSC fraction upon therapeutic inhibition of mTOR.

**Fig. 4:**
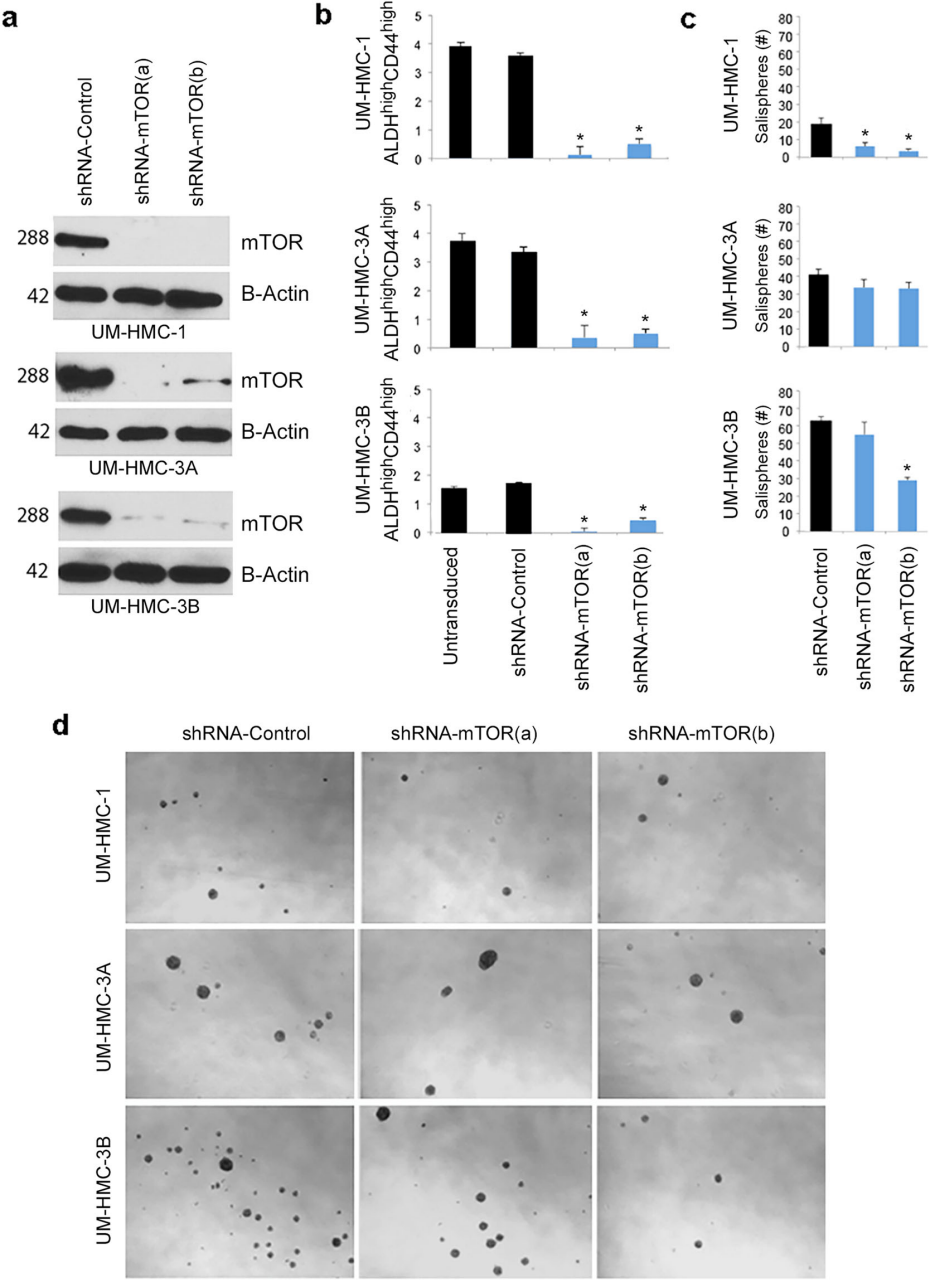
mTOR silencing reduces the fraction of cancer stem-like cells. **a** Western blot analysis to mTOR expression in lysates obtained from mTOR-silenced (shRNA) cells (UM-HMC-1, UM-HMC-3A, and UM-HMC-3B) using two different shRNA sequences (**a**, **b**) or a scrambled sequence vector control (shRNA-control). **b** Graph depicting the fraction of ALDH^high^CD44^high^ cells (UM-HMC-1, UM-HMC-3A, and UM-HMC-3B) identified by flow cytometry in mTOR-silenced cells or vector controls. **c** Graphs depicting the average number of salispheres per well generated by mTOR-silenced cells or vector controls. **d** Representative images of the salispheres evaluated in **c**. Asterisk depicts *p* < 0.05, as determined by one-way ANOVA followed by post-hoc Tukey tests for multiple comparisons between shRNA-Control cells and cells transduced with shRNA-mTOR. Error bars indicate standard deviation (SD). Graphs for impacts of mTOR status on the fraction of ALDH^high^CD44^high^ or number of salispheres depict data from four wells per experimental condition. Experiments were performed three independent times to verify reproducibility of the data.

To verify the data obtained with chemical inhibitors, we used a genetic approach to silence mTOR expression in MEC cells with shRNA-encoding lentiviral vectors. Effectiveness of these vectors to transduce MEC cells was verified by flow cytometry (Supplementary Fig. 10a) and to silence mTOR expression by western blots (Fig. 4a). mTOR-silenced cells consistently showed smaller fraction of CSC cells in three different cell lines and two different shRNA sequences (Fig. 4b and Supplementary Fig. 10b). We also observed that the number of salispheres generated by mTOR-silenced MEC cells is smaller than with vector control cells (Fig. 4c, d).

### Inhibition of mTOR decreases the fraction of CSC in vivo

**Fig. 5:**
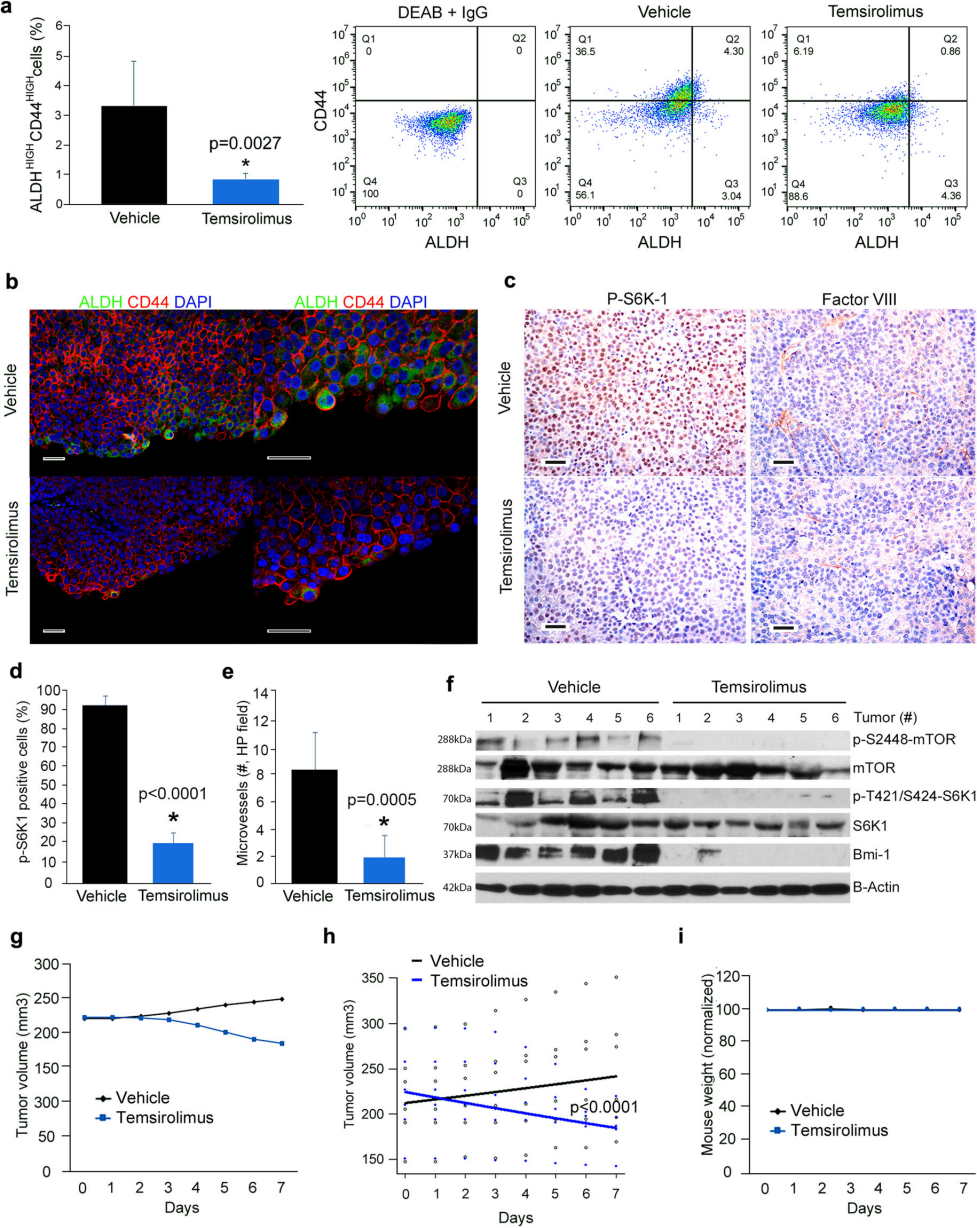
Therapeutic inhibition of mTOR decreases the fraction of cancer stem-like cells in vivo. **a** Graph and flow plots depicting the percentage of ALDH^high^CD44^high^ cells identified by flow cytometry in UM-HMC-3B xenograft tumors retrieved from mice treated with temsirolimus or vehicle control for 7 days. Once tumors (*n* = 6 per experimental condition) reached an average volume of 200 mm^3^, mice received daily intraperitoneal injections of 10 mg/kg temsirolimus or vehicle control. *t*-tests were used to verify statistical significance. **b** Immunofluorescence staining of xenograft tumors after 7-day treatment with 10 mg/kg temsirolimus or vehicle. ALDH1 is stained in green, CD44 is stained in red and DAPI is stained in blue. Scale bars represent 25 µm. **c** Immunohistochemical analysis for p-S6K1 or Factor VIII in xenograft tumors (UM-HMC-3B) retrieved from mice that received temsirolimus or vehicle. Images were taken at 200×. Scale bars represent 25 µm. **d**, **e** Graphs depicting the quantification of p-S6K1-positive cells (**d**) and Factor VIII-positive blood vessels (**e**), as determined by quantification of five microscopic fields per tumor (*n* = 6) by an investigator blinded for experimental conditions. Images were taken at 200×. Scale bars represent 25 µm. **f** Western blot analyses for mTOR, p-S2448-mTOR, S6K1, p-T421/S424-S6K1, and Bmi-1 expression in the whole tumor lysate of each individual tumor (specimen) after 7-day treatment with either 10 mg/kg temsirolimus or vehicle control (*n* = 6). **g** Graph depicting tumor volume after 7 days of treatment with 10 mg/kg temsirolimus or vehicle. **h** Graph depicting regression analysis of tumor volume after 7-day treatment with temsirolimus (blue line) or vehicle control (black line). **i** Graph depicting the average mouse weight during the experimental period. Error bars indicate standard deviation.

To elucidate the impact of therapeutic inhibition of mTOR on MEC CSCs in vivo, we treated mice harboring xenograft tumors with temsirolimus for 7 days. Temsirolimus decreased the fraction of ALDH^high^CD44^high^ cells, when compared with vehicle control (*p* = 0.0027) (Fig. 5a). These data were supported by immunofluorescence analyses showing that the expression of ALDH1 was decreased in temsirolimus-treated tumors (Fig. 5b). In addition, we observed that ALDH1-positive cells undergo apoptosis (cleaved Caspase-3) in xenograft tumors treated with temsirolimus (Supplementary Fig. 11). Immunohistochemical analyses showed that the number of positive p-S6K1 cells is reduced in temsirolimus-treated tumors (*p* < 0.0001) (Fig. 5c, d). Histopathological analysis of these tumors also demonstrated a decrease in microvessel density upon temsirolimus treatment (*p* = 0.0005) (Fig. 5c, e). These results suggest that, in addition to preferential killing of CSCs, temsirolimus may also function by disrupting perivascular niches. Western blots from the xenograft tumor tissues confirmed that temsirolimus is highly effective at inhibiting mTOR and S6K1 phosphorylation in vivo (Fig. 5f). Notably, temsirolimus abrogated the expression of Bmi-1 in vivo (Fig. 5f), confirming results observed in cell lines (Fig. 2). Despite the fact that this in vivo experiment was designed to understand the impact of mTOR inhibition on CSC fraction (and therefore it was a short-term experiment), we observed that tumors treated with temsirolimus regressed (*p* < 0.0001) using a dose that was well tolerated by the mice (Fig. 5g–i).

**Fig. 6:**
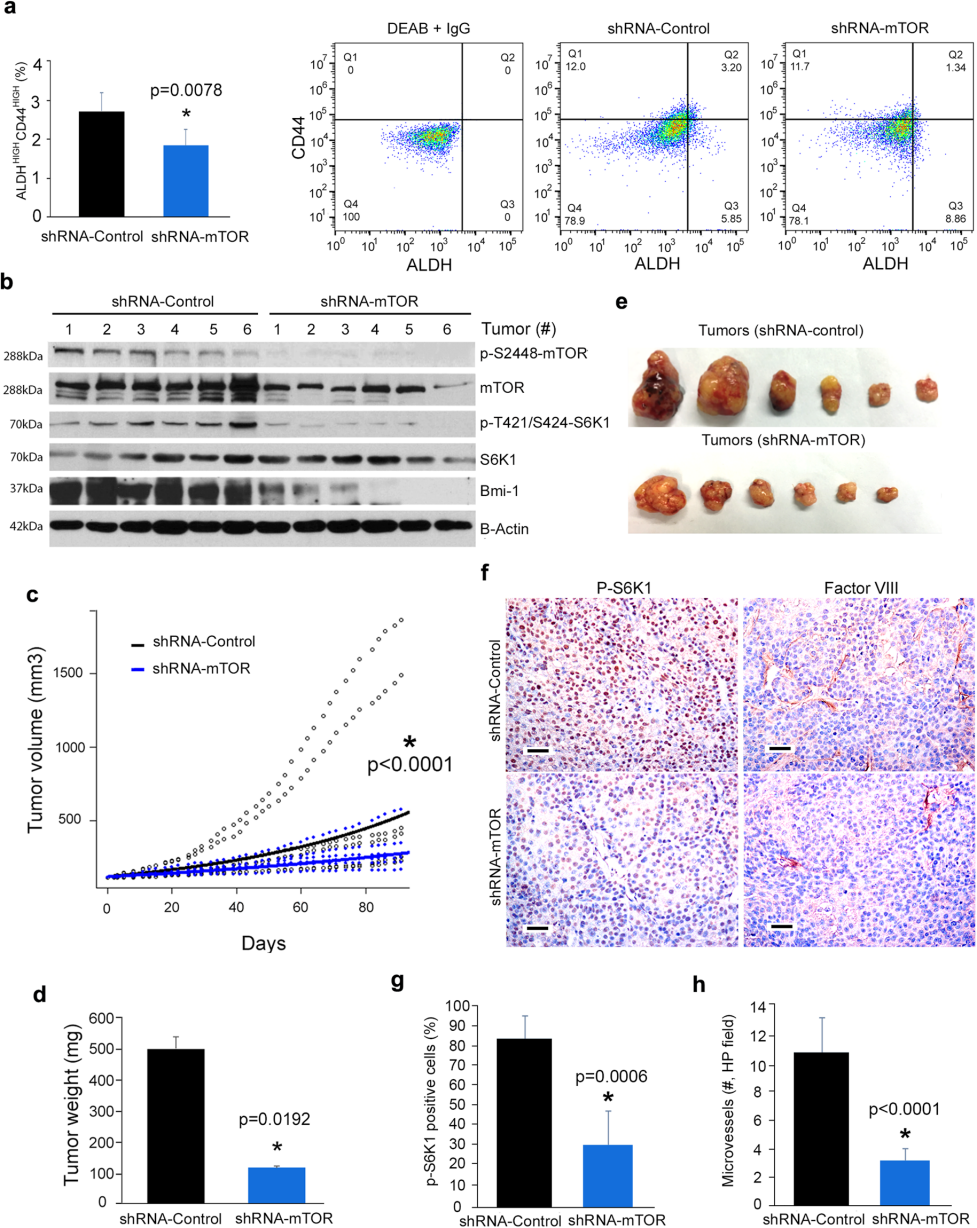
mTOR silencing correlates with reduced fraction of cancer stem-like cells in vivo. **a** Graph and flow plots depicting the percentage of ALDH^high^CD44^high^ cells identified by flow cytometry in tumors generated upon transplantation of mTOR-silenced or scrambled vector control UM-HMC-3B cells (*n* = 6 tumors per experimental condition) 90 days after transplantation of tumor cells into the subcutaneous space of immunodeficient mice. *t*-test was used for statistical analyses of the data. **b** Western blot analyses for mTOR, p-S2448-mTOR, S6K1, p-T421/S424-S6K1, and Bmi-1 expression in lysates from each individual tumor generated by transplantation of mTOR-silenced or vector control cells (UM-HMC-3B) into immunodeficient mice (*n* = 6). **c** Graph depicting regression analysis of volume of tumors generated with mTOR-silenced cells (blue line) or vector control cells (black line). Open black circles represent the volume of each individual tumor, and black line the average volume of tumors generated with shRNA-Control cells. Solid blue circles represent the volume of each individual tumor, and blue line the average volume of tumors generated with shRNA-mTOR cells. **d** Graph depicting final weight of tumors generated by the transplantation of shRNA-mTOR or shRNA-control cells into mice at the end of the experiment (90 days). **e** Image of all xenograft tumors generated by mTOR-silenced or vector control tumor (UM-HMC-3B) cells at the time of mouse euthanasia. **f** Immunohistochemical analyses for p-S6K1 or Factor VIII in mTOR-silenced or vector control xenograft tumors (UM-HMC-3B). **g**, **h** Graphs depicting the quantification of p-S6K1-positive cells (**g**) and Factor VIII-positive blood vessels (**h**), as determined by quantification of five microscopic fields per tumor by an investigator blinded for experimental conditions. Images were taken at 200×. Scale bars represent 25 µm. Error bars indicate standard deviation.

To confirm the data observed with temsirolimus, we transplanted mTOR-silenced MEC (or vector control) cells into immunodeficient mice and followed them up for 90 days. Flow cytometry showed that tumors generated by mTOR-silenced cells had lower fraction of ALDH^high^CD44^high^ cells (*p* = 0.0078) when compared to vector control tumors (Fig. 6a). Western blot results showed significant inhibition of p-S6K1 and Bmi-1 expression in tumors generated by mTOR-silenced cells (Fig. 6b), mimicking in vivo results obtained with chemical inhibitors (Fig. 5f). Tumor growth was slower in xenografts generated by mTOR-silenced MEC cells (*p* < 0.0001), when compared with tumors generated by control cells (Fig. 6c–e). Immunohistochemical analyses revealed a decrease in S6K1 phosphorylation in tumors generated by mTOR-silenced cells (*p* = 0.0006), confirming the results observed in the western blots (Fig. 6b, f, g). Interestingly, a significant decrease in microvessel density was quantified in tumors generated with mTOR-silenced cells when compared to vector control (*p* < 0.0001) (Fig. 6f, h). Collectively, these data demonstrated that mTOR inhibition reduces tumor growth and the fraction of CSCs in MEC.

## Discussion

A stem cell-like population endowed with self-renewal and high tumorigenic potential plays a critical role in the pathobiology of salivary gland mucoepidermoid carcinoma^11^. CSCs are resistant to chemotherapy and are known to drive tumor progression in many tumor types^6–8^. The observation that the mTOR/AKT signaling pathway is constitutively upregulated in MEC CSCs raised the possibility that mTOR inhibitors may eliminate these cells. Indeed, targeted ablation of CSCs might prevent tumor progression and improve long-term patient outcomes^6,7^. Here, we observed a positive correlation between mTOR activation and the long-term clinical outcomes of patients with MEC, which suggests that mTOR might be a potential therapeutic target for these patients.

It is known that mTOR inhibition with temsirolimus causes autophagy of tumor cells in salivary gland adenoid cystic carcinoma^31^. However, the effect of targeting mTOR signaling in MEC CSCs remains unclear. Here, we observed here that MEC CSCs exhibit constitutive activation of mTOR, AKT, and S6K1, as well as expression of Bmi-1, when compared to non-CSC cells. We also observed that ALDH^high^CD44^low^ cells presented lower levels of mTOR pathway activity and Bmi-1 expression than ALDH^high^CD44^high^ cells, and that ALDH^low^CD44^high^ showed mTOR activity and Bmi-1 expression at levels comparable to ALDH^low^CD44^low^ cells. These data suggest that while ALDH might be the principal “driver” of (or has the strongest correlation with) mTOR activation in CSCs, CD44 plays a more modest role regulating these signaling events. Consistent with these findings, we observed that chemical or genetic silencing of mTOR resulted in a decrease in CSC fraction in MEC. These results are consistent with recent studies with PI3K/AKT inhibitors in other tumor types, as colon cancer^32^. Importantly, these data suggest that therapies targeting mTOR function are likely to have a more potent effect in this unique subpopulation of CSCs, which happen to be the most tumorigenic cells in MEC^11^.

It is well known that the mTOR signaling pathway regulates pluripotency and stem cell fate determination, including in embryonic stem cells^33,34^. We observed here that S6K1, a key downstream effector of the mTOR pathway, is a critical step for maintenance and self-renewal of CSCs. PF4708671 is a specific S6K1 inhibitor that does not impact phosphorylation of AKT and mTOR^35^. At low concentrations (not toxic for bulk cells), PF4708671 reduced the CSC fraction, inhibited Bmi-1 expression and salisphere formation. We concluded that S6K1 has a direct impact on the stemness of MEC cells. Although few studies have tested specific inhibition of S6K1, it was demonstrated that S6K1 acts through multiple targets to promote self-renewal and tumor progression in leukemia^36^. These results suggest that S6K1 is an exciting target for future developmental therapeutic studies that aim at ablation of CSCs.

4E-BP1 is a key downstream component of the mTORC1 complex that can act as a tumor suppressor in some cancer types. However, when 4E-BP1 is hyperphosphorylated by mTOR its anticancer effects are prevented^37^. Treatment with mTOR inhibitors releases 4E-BP1, and allow it to have the tumor suppressor effect through inhibition of the translation initiation factor eIF4G, that plays an important role in cell proliferation and tumor formation^37^. We observed that S6K1 and 4E-BP1 phosphorylation are inhibited upon treatment with temsirolimus, which indicates an inhibitory effect of this drug in the mTORC1 of MEC cells.

Our results showed that inhibition of the mTOR pathway preferentially induces apoptosis of CSCs. Indeed, a higher percentage of CSCs undergo apoptosis upon treatment with rapamycin or temsirolimus, when compared to non-CSC cells. We hypothesize that this specificity in induction of CSC apoptosis, is due to the fact that these cells exhibit constitutively active mTOR pathway. Interestingly, platinum-based chemotherapeutic agents not only do not eliminate CSCs in MEC^38^ and in head and neck squamous cell carcinomas (HNSCC)^39^, but also enable their self-renewal and accumulation in the tumor^39^. The results that we observed with blockade of mTOR signaling in MEC are consistent with data from other cancer types^40–42^.

We observed that mTOR inhibition was correlated with inhibition of AKT phosphorylation in MEC cells, which was also observed in HNSCC cells^43^. It is known that rapamycin universally inhibits mTORC1^18^ via the rapamycin-FKBP12 interaction^22^. Studies have also shown that chronic exposure to rapamycin promotes inhibition of free mTOR molecules, which inhibits the formation of new mTORC2 complexes in some tumor types^44,45^. Notably, mTORC2 typically has a positive feedback effect on AKT, thus enhancing cell survival^30^. The Sabatini laboratory used rapamycin in multiple cell lines to evaluate the effects of mTOR inhibition on downstream targets, including AKT and Rictor^45^. The authors concluded that not all cells respond in the same manner to rapamycin, and suggested the possibility that cells with high proliferation rate tend to be more sensitive to mTOR inhibition. For example, rapamycin treatment inhibited AKT phosphorylation at the S473 residue in some cell lines (e.g., PC3, U937, and Jurkat), but not in others (e.g., HeLa, 293T, and LNCap). In rapamycin-sensitive cells, inhibition of mTORC2 assembly and AKT activation leads to a decrease in cell survival, while this effect is not observed in rapamycin-insensitive tumor cells^18^. In light of these results, one predicts that the MEC cells tested here belong to the group of tumor cells that is sensitive to mTOR inhibition, which would explain the consistent downregulation of p-S473-AKT with two different mTOR inhibitors (rapamycin, temsirolimus) in three independent cell lines observed here.

While our shRNA-based strategy was effective at silencing mTOR expression and was associated with a decrease in the fraction of CSCs in three MEC cell lines, the effect of genetic downregulation of mTOR on salisphere formation was not as consistent. Despite the fact that we selected these cells with puromycin for several days, we know that shRNA-mediated gene silencing might not be equally effective in every cell. Considering the fact that it takes only one CSC to generate a new sphere, it is possible that a small number of cells escaped shRNA-mediated mTOR silencing and generated salispheres. Nevertheless, we observed an overall trend for decreased number of salispheres upon mTOR silencing in the MEC cells, corroborating the results obtained with the chemical inhibitors.

**Fig. 7:**
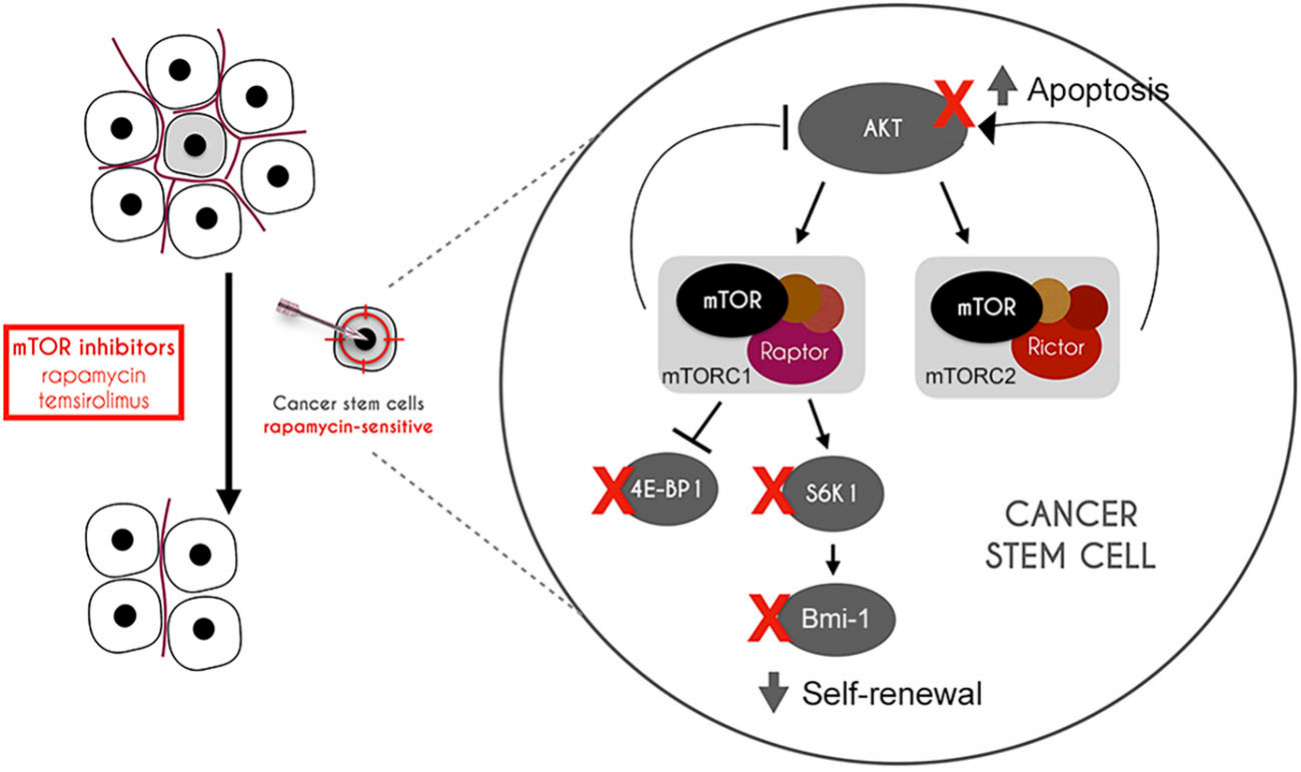
Schematic view of the proposed mechanism underlying the effect of therapeutic inhibition of mTOR on cancer stem-like cells in mucoepidermoid carcinoma. As cancer stem-like cells exhibit constitutive activation of the mTOR pathway and are addicted to pro-survival signaling mediated by this pathway, effective inhibition of mTOR results in inhibition of Bmi-1 (self-renewal) and AKT activity. The net effect of these events is a decrease in the fraction of cancer stem-like cells in salivary gland mucoepidermoid carcinoma upon therapeutic inhibition of mTOR signaling.

In vivo experiments showed that therapeutic or genetic blockade of mTOR signaling causes inhibition of S6K1 phosphorylation, decreased Bmi-1 expression, and reduction in CSC fraction. We also observed that inhibition of mTOR showed signs of tumor regression, despite the fact that this study was short-term (7 days) and not designed to measure effects on tumor volume (focus was on CSC effects). Interestingly, we observed a reduction in tumor microvessel density upon mTOR inhibition, which has been reported in other tumor models^46,47^. Thus, therapeutic inhibition of mTOR reduces the fraction of CSCs via a direct effect (i.e., induction of apoptosis) and may also have an indirect effect via disruption of their stem cell niche, which is clearly important for maintenance of these cells in cancer^48^. We conclude that therapeutic blockade of mTOR is sufficient to inhibit cancer stemness in MEC by mediating selective ablation of CSCs (Fig. 7). Collectively, these results suggest that patients with salivary gland mucoepidermoid carcinoma might benefit from therapeutic inhibition of the mTOR pathway.

## Materials and methods

### Human mucoepidermoid carcinoma cells

Human salivary gland mucoepidermoid carcinoma cells lines (UM-HMC-1, UM-HMC-3A, and UM-HMC-3B) generated and characterized in our laboratory^49^ were maintained in salivary gland culture media consisting of Dulbecco’s Modified Eagle’s Medium (DMEM; Thermo Scientific; Rockford, IL, USA) supplemented with 10% FBS (Gibco), 1% l-glutamine (Gibco), 1% penicillin and streptomycin (Thermo Scientific), 20 ng/ml Epidermal Growth Factor (EGF; MilliporeSigma; Burlington, MA, USA), 400 ng/ml hydrocortisone (MilliporeSigma) and 5 µg/ml Insulin (MilliporeSigma). The MEC cell lines have been continuously STR-profiled and matched to the original patient tumor. Primary human dermal microvascular endothelial cells (HDMEC; Lonza, Walkersville, MD, USA) were cultured using the endothelial growth medium for microvascular cells (EGM2-MV; Lonza). All cell lines were negative for mycoplasma contamination.

### Human subjects

Tumor samples from 17 patients diagnosed with MEC and their clinical follow-up data without patient identifiers (Supplementary Fig. 1b) were provided by the Pathology department from the University of Campinas (Campinas, São Paulo, Brazil) and by the University Center of Pará (Belém, Pará, Brazil). Ethical approval for access to these histological sections was obtained through the Human Subjects Ethics Committee of the School of Dentistry, UNICAMP and University of São Paulo, Brazil. All patients signed an informed consent form authorizing the use of their clinical data and tissue specimens for research.

### Cytotoxicity assays

Sulforhodamine B (SRB) assays were performed to evaluate the effect of inhibitors of the mTOR signaling pathway on MEC cell density in tissue culture plates. Cells (10^3^ cells per well) were seeded in 96-well plates, allowed to attach overnight and exposed to increasing concentrations of the following reagents: AKT inhibitors, buparlisib (SelleckChem; Houston, TX, USA), LY294002 (MilliporeSigma); mTOR inhibitors, rapamycin (MilliporeSigma), temsirolimus (MilliporeSigma); or S6K1 inhibitor, PF4708671 (SelleckChem) for 24–96 h. Cells were fixed with 10% cold trichloroacetic acid for 1 h at 4 °C. Then, viable cells were stained with 0.4% SRB dye (MilliporeSigma; Burlington, MA, USA) for 30 min. To remove excess of unbound dye plates were washed with 1% acetic acid. 10 mMol/l tris base was used to solubilize stained cells and plates were read in a microplate reader at 560 nm (GENios, Tecan; Männedorf, Switzerland). Data were obtained from quadruplicate wells/condition. Here, and throughout this work, three independent in vitro experiments were performed to verify reproducibility of data.

### Western blots

Protein lysates from UM-HMC cells and dissociated xenograft MEC tissues were prepared using 1% Nonidet P-40 (NP-40) lysis buffer and loaded onto 9–12% SDS-PAGE gels. Membranes were incubated overnight at 4 °C with 1:1000 dilution of the following antibodies: rabbit antihuman p-AKT Ser 473 (Cell Signaling, Danvers, MA, USA), mouse antihuman AKT (Cell Signaling), rabbit antihuman p-Rictor Thr1135 (Cell Signaling), mouse antihuman Rictor (Santa Cruz Biotechnology; Dallas, TX, USA), rabbit antihuman p-mTOR Ser 2448 (Santa Cruz), rabbit antihuman mTOR (Cell Signaling), mouse antihuman p-4E-BP1 Ser 65 (Santa Cruz), mouse antihuman p-4E-BP1 (Santa Cruz), rabbit antihuman p-S6K1 Thr 421/Ser 424 (Santa Cruz), rabbit antihuman S6K1 (Cell Signaling), rabbit antihuman Bmi-1 (Cell Signaling). As surrogate for loading control we used Western blot performed with 1:100,000 antihuman β-actin antibody (Santa Cruz). Membranes were exposed to 1:10,000 affinity-purified secondary antibodies conjugated with horse-radish peroxidase (Jackson Laboratories; West Grove, PA, USA). Immunoreactive proteins were visualized by SuperSignal West Pico chemiluminescent substrate (Thermo Scientific).

### Flow cytometry

Cells were filtered using 5 ml polystyrene round-bottom tube with cell strainer caps (BD Biosciences; Bedford, MA, USA). 7-aminoactinomycin (7-AAD; BD Biosciences) was used to eliminate dead cells. ALDH activity was quantified using the Aldefluor kit (StemCell Technologies; Cambridge, MA, USA). Single cell suspensions of 2 × 10^6^ cells/ml were prepared and incubated with 5 μl Aldefluor substrate, or 5 μl diethylaminobenzaldehyde (DEAB, ALDH activity inhibitor) for 40 min at 37 °C. Then, cells were washed and exposed to 1:100 anti-CD44 (APC; BD Biosciences) for 30 min at 4 °C. When required, cells were sorted according to ALDH activity and expression of CD44. Alternatively, cells were stained with combination of AldeRed 588-A (Cayman; Ann Arbor, MI, USA) and CD44 violet (V450, BD Biosciences) for experiments with GFP-transfected cells. For apoptosis studies, cells were exposed to rapamycin, temsirolimus, cisplatin (Teva; Cincinnati, OH, USA) or paclitaxel (Fresenius Kabi; Grand Island, NY, USA) for 24 h. Cells were incubated with Aldefluor substrate (StemCell Technologies), CD44 violet (V450, BD Biosciences), and 1:100 Annexin V (APC; BD Biosciences) for 20 min at room temperature. Cells treated with DMSO were used as control for the Annexin V studies.

### Salisphere assay

Non-adherent spheroids derived from mucoepidermoid carcinoma cells (salispheres) were cultured in ultralow attachment 6-well plates (Corning; Corning, NY, USA) for 10 days with DMEM-F12 (Invitrogen, Carlsbad, CA, USA) supplemented with 1% N-2 supplement (Invitrogen), 1% GlutaMAX (Invitrogen), 1% penicillin/streptomycin (Invitrogen), 1 μM dexamethasone (MilliporeSigma), 10 μg/ml insulin (MilliporeSigma), 20 ng/ml EGF (MilliporeSigma), and 20 ng/ml basic fibroblast growth factor (bFGF; MilliporeSigma). For in vitro passaging, salispheres were exposed to 0.25% trypsin (MilliporeSigma) for 5 min, and then mechanically dissociated. A neutralizing solution (TNS; Lonza) was used to neutralize trypsin. Cells were counted and then added to new 6-well ultralow attachment plates (Corning; Corning, NY, USA). Colonies of 50 cells or more were considered salispheres.

### mTOR silencing in UM-HMC cells

HEK-293T cells were transiently transfected with the lentiviral packaging vectors psPAX2, pMD2G, and shRNAs for mTOR [two sequences, shRNA-mTOR(a) or shRNA-mTOR(b)] or scramble sequence vector control (shRNA-control) (Vector Core, University of Michigan) using the calcium phosphate method. UM-HMC cells were infected with supernatants containing lentiviruses and selected with 1 µg/ml of puromycin (MilliporeSigma) for at least 1 week. Knockdown of mTOR was verified by western blot and flow cytometry for GPF expression, as described above.

### Mucoepidermoid carcinoma xenograft tumors

Highly porous poly-l-lactic acid scaffolds (6 × 6 × 1 mm) were prepared and seeded with 700,000 UM-HMC-3B cells together with 300,000 primary HDMEC cells (Lonza) in a 1:1 mix of growth factor-reduced (Matrigel; Corning) and EGM2-MV (Lonza), as we described^50^. Two scaffolds were implanted in the subcutaneous space of the dorsal region of each 7–8-weeks-old female severe combined immunodeficient mouse (CB.17. SCID; Charles River Laboratory, Mattawan, MI, USA). Tumors were measured weekly and tumor size was calculated using the formula: volume (mm^3^) = *L* × *W*2/2 (*L*, length; *W*, width). When the average tumor volume reached 200 mm^3^, mice (*n* = 6) were randomly allocated to the following experimental conditions: daily intraperitoneal injection of 10 mg/kg temsirolimus (SelleckChem) or vehicle control (5% polyethylene glycol-200 and 0.5% tween 80 in water) for 7 days. For tumor growth studies, sample size was determined from data published by our laboratory using the same experimental approaches^30^. To verify the impact of mTOR silencing, 700,000 UM-HMC-3B mTOR-silenced cells or UM-HMC-3B transduced with shRNA vector were seeded together with 300,000 HDMEC cells per scaffold, as described above (*n* = 6). Tumors were measured three times/week for 90 days, when first tumors reached 1 cm^3^. For both experiments, mice were euthanized, tumors were removed, and divided in thirds for the following experiments: western blot, immunohistochemistry and flow cytometry. The first 1/3 was fixed overnight in 10% buffered formalin (Thermo Fisher) at 4 °C and processed for immunohistochemistry; the second 1/3 was exposed to 1% Nonidet P-40 (NP-40) lysis buffer to prepare protein lysates for western blots. And finally, the third 1/3 of the tumors was minced in small pieces and digested in 1× collagenase–hyaluronidase (StemCell Technologies) at 37 °C for 60 min. Cells were passed through a 40-μm sieve (Thermo Fisher) and salivary gland media with fetal bovine serum (FBS, Invitrogen) was added to cells. Red blood cells were lysed for 1 min using AKC lysis buffer (Invitrogen). Then, single cell tumor cell suspensions were stained for flow cytometry, as described above. Sample size was calculated based on power calculations using similar studies published by our laboratory as reference^51^. The care and treatment of experimental animals was in accordance with an University of Michigan IACUC-approved protocol (PRO00007199).

### Immunohistochemistry and immunofluorescence

Tissue sections were incubated at 60 °C, deparaffinized in xylene and rehydrated in ethanol. Citrate Buffer Antigen Retrieval kit (Dako; Troy, MI, USA) was used for antigen retrieval. Tissues were permeabilized by incubation with 0.1% Triton-x100 (MilliporeSigma) for 20 min, 3% hydrogen peroxide for 20 min, and background sniper (Biocare Medical; Pacheco, CA, USA) for 20 min. Immunohistochemistry slides were incubated overnight at 4 °C with 1:50 antihuman p-mTOR antibody (Santa Cruz Biotechnology), 1:100 antihuman p-S6K-1 antibody (Santa Cruz Biotechnology), or 1:50 antihuman factor VIII antibody (Thermo Scientific). Sections were washed twice for 10 min with phosphate buffered saline (PBS) and then incubated with MACH 3 Probe (Biocare Medical) for 20 min. Sections were washed again twice in PBS for 10 min and incubated with MACHI 3 HRP polymer for 20 min. Finally, sections were stained with chromogen 3,3-diaminobenzidine (DAB), counterstained with Mayer hematoxylin, dehydrated in ethanol and mounted. Immunofluorescence sections were co-incubated with 1:100 monoclonal rabbit antihuman ALDH1 antibody (Abcam; Cambridge, MA, USA) and mouse 1:200 antihuman CD44 antibody (Abcam) or cleaved Caspase-3 Asp175 (Cell Signaling) overnight at 4 °C temperature. The next day, sections were washed with PBS twice for 10 min and incubated with Alexafluor 488 (AntiRabbit; Invitrogen) for 20 min. Then, sections were incubated with Alexafluor 594 (AntiMouse; Invitrogen) for 20 min. Nuclei were stained with DAPI. Slides were visualized under light or fluorescence microscopy (Leica DM 5000B), and images were taken with Q-Capture Pro 7 (Q-imaging, Tucson, AZ, USA). Images were quantified by an experienced oral pathologist blinded for experimental conditions. Ten random fields per slide at 400× magnification were evaluated and the percentage of positive cells was quantified using ImageJ (NIH) software.

### Statistical analysis

Data from all in vitro experiments were analyzed by one-way ANOVA, followed by post-hoc Tukey tests for multiple comparisons or Student’s *t*-test. For in vivo experiments, mixed effect linear regression was employed to allow for interpretation of repeated measurements on each tumor. The tumor volume data was log-transformed to account for exponential growth. The model fixed effects included time, tumor starting size, and either treatment or shRNA condition. Random effects included mouse. We assumed an autoregressive correlation structure so that more proximate time values are assumed to be more correlated. Prediction curves were generated from the regression model. Analysis was performed using the “gls” package in the software program R, version 3.1.0. Statistical comparison of mean cell percent p-S6K1 positive and mean cell percent mTOR positive between individual patients experiencing recurrence or death, and those surviving were each computed using Mann–Whitney *U*-test. Correlation between tumor grade and immunohistochemical components was computed using Spearman’s rank-correlation test. All computations were performed using the statistical software R 3.1.0. Significance was determined at *p* < 0.05.

## Supplementary information

Supplementary Information (suppl. figures)

Supplementary Figure 1

Supplementary Figure 2

Supplementary Figure 3

Supplementary Figure 4

Supplementary Figure 5

Supplementary Figure 6

Supplementary Figure 7

Supplementary Figure 8

Supplementary Figure 9

Supplementary Figure 10

Supplementary Figure 11

## Supplementary information

**Supplementary Information** The online version contains supplementary material available at (10.1038/s41419-021-03391-7).
