# Supplementary material for: Survival of salivary gland cancer stem cells requires mTOR signaling
Source: Cell Death Dis. 2021 Jan 21;12(1):108. doi: 10.1038/s41419-021-03391-7 (PMC7820616; doi:10.1038/s41419-021-03391-7)
Supplement: Supplementary file 1 — Supplementary Information (suppl. figures) [file 41419_2021_3391_MOESM1_ESM.docx]

**Supplementary figures legends**

**Supplementary figure 1. a** Representative images of immunohistochemistry staining for p-mTOR or p-S6K1in tissue samples retrieved from patients with salivary gland mucoepidermoid carcinoma. Scale bars represent 25 µm. **b** Table depicting clinical findings and the average percentage of p-mTOR-positive and p-S6K1-positive cells per tumor, as determined by evaluation of stained sections by an experienced oral pathologist.

**Supplementary figure 2**. Graphs depicting time- and dose-dependence assays for the effect of buparlisib (AKT inhibitor); rapamycin and temsirolimus (mTOR inhibitors); and PF4708671 (S6K1 inhibitor) on the viability of UM-HMC-1, UM-HMC-3A and UM-HMC-3B cells, as determined by the SRB assay. Data were normalized against vehicle control and represent 4 wells/condition. Error bars indicate standard deviation (SD). Experiments were performed 3 independent times to verify reproducibility of the data.

**Supplementary figure 3.** **a** and **b** Representative flow plots (from data presented in Figure 2a) depicting flow cytometry for ALDH activity and CD44 expression in UM-HMC-1, UM-HMC-3A and UM-HMC-3B cells after exposure to increasing concentrations of AKT inhibitors, *i.e.* 0.2-20 μM buparlisib (**a**) or 0.2-20 μM LY2940002 (**b**) or vehicle control.

**Supplementary figure 4. a** and **b** Representative flow plots (from data presented in Figure 2b) depicting flow cytometry for ALDH activity and CD44 expression in UM-HMC-1, UM-HMC-3A and UM-HMC-3B cells after exposure to increasing concentrations of mTOR inhibitors, 0.2-20 ng/ml rapamycin (**a**) or 0.2-20 ng/ml temsirolimus (**b**) or vehicle control.

**Supplementary figure 5.** Representative flow plots (from data presented in Figure 2c) depicting flow cytometry for ALDH activity and CD44 expression in UM-HMC-1, UM-HMC-3A and UM-HMC-3B cells after exposure to increasing concentrations of S6K1 inhibitor (2-200 nM PF4708671) or vehicle control.

**Supplementary figure 6. a** Graph depicting the average number of salispheres per well of UM-HMC-1, -3A and -3B cell lines after exposure to 20 µM buparlisib, 20 µM LY2940002, 2 ng/ml rapamycin, 2 ng/ml temsirolimus, 200 nM PF4708671 or vehicle control. Different low-case letters indicate significant differences among groups (*P*<0.05), as determined by one-way ANOVA followed by post-hoc Tukey tests for multiple comparisons. Error bars indicate standard deviation (SD). Graphs for impacts of treatment on the number of salispheres depict data from 4 wells per experimental condition. Experiments were performed 3 independent times to verify reproducibility of the data. **b** Representative images of primary and secondary salispheres generated by UM-HMC-1, UM-HMC-3A or UM-HMC-3B cells after exposure to 20 µM LY2940002, 2 ng/ml rapamycin, 2 ng/ml temsirolimus, 200 µM PF4708671 or vehicle control (40x magnification).

**Supplementary figure 7. Flow cytometry gating strategy for evaluation of early apoptotic events (Annexin V) in cancer stem cells (ALDH^high^CD44^high^). a** Exclusion of cell debris and necrotic cells through 7-AAD staining**. b** Analysis of Annexin V expression. **c** Analysis of MEC cells for ALDH activity and CD44 expression. DEAB was used to control for ALDH substrate. IgG was used as control for CD44 antibody. **d** Final flow plot where the fraction of apoptotic cells per quadrant (ALDH activity, CD44 expression) was determined. Viable cells are represented in dark gray while early apoptotic cells (*i.e.* high expression of Annexin V) are shown in red. Total number of cells per quadrant are expressed in the top of each quadrant.

**Supplementary figure 8. mTOR inhibitors are more effective at inducing apoptosis of cancer stem-like cells than bulk tumor cells. a** Flow plots for ALDH activity, CD44 and Annexin V expression in UM-HMC-1 cell line after 24 hours of treatment with increasing concentrations of rapamycin, temsirolimus, cisplatin or paclitaxel analyzed by flow cytometry. Viable cells are represented in dark gray while early apoptotic events (*i.e.* high expression of annexin V) are shown in red. **b** Graphs depicting the percentage of apoptotic cells according to ALDH activity and CD44 expression, *i.e.* ALDH^high^CD44^high^, ALDH^high^CD44^low^, ALDH^low^CD44^high^ ALDH^low^CD44^low^ after treatment with rapamycin, temsirolimus, cisplatin or paclitaxel. **c** Graph depicting the percentage of ALDH^high^CD44^high^ cells identified by flow cytometry in MEC tumor cells (UM-HMC-1) treated with increasing concentrations of rapamycin, temsirolimus, cisplatin or paclitaxel. Different low-case letters indicate significant differences among groups (*P*<0.05), as determined by one-way ANOVA followed by post-hoc Tukey tests for multiple comparisons. Error bars indicate standard deviation (SD). Graphs for impacts of treatment on the fraction of ALDH^high^CD44^high^ or percentage of apoptotic cells depict data from 4 wells per experimental condition. Experiments were performed 3 independent times to verify reproducibility of the data.

**Supplementary figure 9. mTOR inhibitors are more effective at inducing apoptosis of cancer stem-like cells than bulk tumor cells. a** Flow plots ALDH activity, CD44 and Annexin V expression in UM-HMC-3A cells after 24 hours of treatment with increasing concentrations of rapamycin, temsirolimus, cisplatin or paclitaxel analyzed by flow cytometry. Viable cells are represented in gray while early apoptotic cells (*i.e.* high expression of annexin V) are shown in red. **b** Graphs depicting the percentage of apoptotic cells according to ALDH activity and CD44 expression, *i.e.* ALDH^high^CD44^high^, ALDH^high^CD44^low^, ALDH^low^CD44^high^ ALDH^low^CD44^low^ after treatment with rapamycin, temsirolimus, cisplatin or paclitaxel. **c** Graph depicting the percentage of ALDH^high^CD44^high^ cells identified by flow cytometry in tumor cells (UM-HMC-3A) treated with increasing concentrations of rapamycin, temsirolimus, cisplatin or paclitaxel. Different low-case letters indicate significant differences among groups (*P*<0.05), as determined by one-way ANOVA followed by post-hoc Tukey tests for multiple comparisons. Error bars indicate standard deviation (SD). Graphs for impacts of treatment on the fraction of ALDH^high^CD44^high^ or percentage of apoptotic cells depict data from 4 wells per experimental condition. Experiments were performed 3 independent times to verify reproducibility of the data.

**Supplementary figure 10. a** Representative flow plots (from data presented in Figure 4a) depicting flow cytometry for GFP staining in MEC cells (UM-HMC-1, UM-HMC-3A, UM-HMC-3B) transduced with shRNA-control, shRNA-mTOR(a) and shRNA-mTOR(b) as well untransduced control cells. **b** Representative flow plots (from data presented in Figure 4b) depicting flow cytometry for ALDH and CD44 staining in MEC cells transduced with shRNA-control, shRNA-mTOR(a) and shRNA-mTOR(b) or untransduced control cells.

**Supplementary figure 11.** Immunofluorescence staining of xenograft tumors generated with UM-HMC-3B cells that were treated with temsirolimus or vehicle for 7 days. ALDH1 is stained in green, cleaved caspase-3 is stained in red, and DAPI in blue. The first and second large images (top) represent tumors from the vehicle control group and show that ALDH-positive cells were not undergoing apoptosis, and that the few cells undergoing apoptosis (cleaved Caspase-3) were ALDH-negative. The third large image (top) represents a tumor from the temsirolimus-treated group depicting an ALDH-positive apoptotic cell (*i.e.* positive for cleaved-Caspase-3). The smaller inserts underneath each image depict each individual immunofluorescence staining (before merging the images). Scale bars indicate relative size.
